# Valuing health as development: going beyond gross domestic product

**DOI:** 10.1136/bmj.k4371

**Published:** 2018-10-23

**Authors:** Victoria Y Fan, David E Bloom, Osondu Ogbuoji, Klaus Prettner, Gavin Yamey

**Affiliations:** 1 Office of Public Health Studies, Myron B Thompson School of Social Work, University of Hawai′i at Mānoa, Honolulu, USA; 2 Harvard TH Chan School of Public Health, Boston, MA, USA; 3 Duke Global Health Institute, Duke University, Durham, NC, USA; 4 University of Hohenheim, Stuttgart, Germany

## Abstract

GDP per capita is a narrow, inadequate metric for capturing the true, full value of health investments, say **Victoria Fan and colleagues**

Gross domestic product (GDP), which represents the value of goods and services that are produced in a year in a given country, is the standard metric for assessing economic growth and development. Research on the link between health and economic growth has, therefore, traditionally looked at whether investing in health increases GDP (or, conversely, whether uncontrolled diseases such as malaria reduce GDP). Changes in GDP per capita can capture the “instrumental” effects of better health—that is, the way in which better health generates greater income through factors such as higher worker productivity. But GDP is a narrow, inadequate metric for capturing the true, full value of health investments.^1^ ^2^

Pronouncements on the state of the nation’s economy regularly make headline news. In the United States, for example, politicians and reporters discuss the Bureau of Economic Analysis’s quarterly releases of the nation’s GDP as an indication of the country’s “great” development and direction.^3^ This phenomenon is not restricted to the US but is seen worldwide.^4^

Fortunately, people are realising the inadequacy of GDP per capita as a measure of development and wellbeing.^5^ Economists recognise the importance of GDP per capita as an aggregate measure of production, but they have long argued that it does not capture important aspects of life that we all value, including health, education, distributive fairness, and the quality of the environment. The highest profile critique of GDP in recent years was the 2009 report of the Stiglitz-Sen-Fitoussi commission (the Commission on the Measurement of Economic Performance and Social Progress).^2^ The commission noted that, although GDP is mainly a measure of market production, it is often treated as a measure of economic wellbeing and that “conflating the two can lead to misleading indications about how well-off people are and entail the wrong policy decisions.”

Here we ask, why does GDP still dominate discussions about a country’s state of development? What are the flaws in using GDP to measure a nation’s development? And what should be the primary indicator by which to measure a nation’s development and direction?

## Why GDP dominates our discourse

GDP, GDP per capita, and their growth rates are popular in policy and media circles for several reasons. They are important indicators of the current state of the economy. They are available for a long historic period of time, allowing for analysis of an economy’s evolution over time and for comparisons between countries.

GDP’s appealing simplicity stands in contrast to the many hundreds of indicators that are now used in development—a “mashup of indices”^6^ in which multiple markers are combined into a single composite. Combining GDP with other factors results in the loss of information on key indicators of development that are independent of income.

The modern conception of GDP is credited to the economist Simon Kuznets, who proposed a measure to capture the productive capacity of individuals, companies, and the government in a 1937 report to the US Congress.^7^ But Kuznets warned against using GDP as a welfare measure, stating that “distinctions must be kept in mind between quantity and quality of growth.”^8^ Over time, however, GDP became widely used as a measure of economic development, as reflected in reports from the International Monetary Fund, the World Bank, the economic bureaus of many countries, and others.^9^ Economist Robert Lucas and others emphasised that economic development was the (narrow) process in which income per capita increases, driven by the accumulation of physical capital, “human capital” (through schooling, for example), and technological progress.^10^ ^11^

## Flaws in using GDP per capita to assess a nation’s development

As Robert F Kennedy said in 1968, GDP fails to measure “the health of our children, the quality of their education, or the joy of their play. It does not include the beauty of our poetry or the strength of our marriages, the intelligence of our public debate or the integrity of our public officials . . . it measures everything in short, except that which makes life worthwhile.”^12^

Many others, including economists, have also pointed out the flaws and inadequacies of GDP. One representative view is that of Nobel laureate and economist Amartya Sen, who argued that economic development is the broad process by which the human condition is improved and through which people’s choices are enlarged. In his book *Development as Freedom*, Sen argued that “Development can be seen . . . as a process of expanding the real freedoms that people enjoy.”^13^ He further argued that focusing on human freedoms “contrasts with narrower views of development, such as identifying development with a growth of gross national product (GNP), or with a rise in personal income, or with industrialisation, or with technological advance, or with social modernisation.”

The Stiglitz-Sen-Fitoussi commission raised the shortfalls of GDP as a measure of “economic performance and social progress.”^2^ The commission indicated that it was time “for our measurement system to shift emphasis from measuring economic production to measuring people’s wellbeing” and went on to define wellbeing as a multidimensional concept that includes health and education.

Furthermore, growing inequality in almost all countries worldwide—emphasised in the US by the Occupy Wall Street movement with its slogan “we are the 99%” (the other 1% are the wealthy)^14^ and in the UK by the Equality Trust (www.equalitytrust.org.uk)—has shown another limitation of GDP. In light of such inequality, the emphasis on GDP has shifted to recognise that averages alone are inadequate and that considerations of distribution are valuable. Examples of inequality measures are the Gini coefficient and the total income held by the top 1% of the population compared with the 99%.^15^

In summary, GDP is limited in measuring only marketed goods and services, does not capture inequality, and disregards several aspects of wellbeing such as environmental quality and the utilitarian values of education and health (box 1).

Box 1 **The wealth and health of two countries**

Consider this comparison of Bangladesh and Zambia. The two countries have a similar GDP per capita ($3720 (£2900; €3200) and $3860, respectively, in 2018, adjusted for purchasing power), yet the countries’ health indicators differ vastly. A baby born in Bangladesh will live 73 years on average, whereas a baby born in Zambia will live for an average of 60 years. Using GDP per capita alone fails to capture the inherent or “intrinsic” value of those 13 extra years of life in Bangladesh. Those additional years have an economic value that goes beyond productivity. As Bloom and colleagues put it: “A country whose citizens enjoy long and healthy lives clearly outperforms another with the same GDP per capita but whose citizens suffer much illness and die sooner.”^3^

## Alternative measures of progress

Clearly, GDP should not be used alone but alongside complementary indicators. Economists have proposed several alternatives and continue to do so. In his influential 2002 paper, Nordhaus—one of the two winners of the 2018 Nobel prize in economics—proposed an alternative called “health income,” which incorporates improvements in health status into measures of national income.^5^ More recently, Jones and Klenow proposed a measure of the welfare of a country’s population that combines data on consumption (the use of goods and services by households), leisure, inequality, and mortality.^16^ The UK Office of National Statistics routinely publishes a national wellbeing measure.^17^ But perhaps the best known is the Human Development Index (HDI), developed by the United Nations Development Programme (UNDP). The HDI measures living standards across three major dimensions: life expectancy at birth, education, and GDP per capita. In devising this measure, the UNDP placed health and education on an equal plane with the economy.^18^ ^19^

But the HDI has not seen much traction among multilateral agencies. It is more difficult to compute than GDP (by virtue of combining GDP with two other indicators), it is not useful as a time series indicator because of its limited availability, its units are not easily understood, and it has other numeric limitations (the HDI ranges only from 0 to 1). UNDP also developed several other indices, such as the multidimensional poverty index and the gender inequality index.^20^ However, to the best of our knowledge, few if any countries put much emphasis on these indices in their quarterly government reports. 

Another alternative to GDP in valuing health investments—an approach borrowed from environmental economics—is the “value of life years” (VLY) approach (box 2).^21^ In its *Global Health 2035* report, the Lancet Commission on Investing in Health used the VLY approach, arguing that it gave “a more accurate and complete picture of health’s contribution to a nation’s economic wellbeing” than using GDP alone.^21^ The commission estimated the economic returns of investing in a “grand convergence”—a universal reduction in avoidable deaths from infections and maternal and child health conditions. Using VLY, it estimated that every $1 invested in achieving grand convergence would yield $9-20, a remarkable rate of return. Jim Kim, president of the World Bank, noted that the commission provided “further proof that improvements in human survival have economic value well beyond their direct links to gross domestic product.”^22^

Box 2 **Health or material goods—which would you choose?**

William Nordhaus poses the following question: Imagine that you have to choose between the improvements in material goods since the 1950s and the improvements in health during the same period. In other words, you have to choose between today’s cell phones, computers, wi-fi, roads, air travel, and other material benefits versus an extra 11 years of life expectancy without those goods.

Most people would choose the second option, indicating that people place a high value on living longer, a value that is not connected to material gain. The concept of the “value of life years” attempts to capture the intrinsic value of living longer.

Nevertheless, the VLY, and the related concept of the value of a statistical life, are often subjective measures, and they strongly depend on who is studied when estimating their values. Estimates of the monetary value of VLY come from willingness-to-pay studies and various other study methodologies, such as standard gamble, time trade-offs, and discrete choice experiments.^23^ Approaches include: asking people how much they would be willing to pay to reduce their risk of dying; estimating their willingness to pay to reduce this risk, based on their consumption choices; and observing how much money people actually get paid for risky occupations.^23^ In other words, the additional life expectancy is “monetized” by asking people or inferring what an extra day is worth to them. The results differ based on who is interviewed. A person who is very risk averse, for example, would pay a lot more to avoid risks and would gamble much less than a person who is risk loving, and individuals from countries with lower GDP per capita tend to report a lower willingness to pay.^24^

In creating any composite measure, two key but challenging questions arise: what measures should be included and what is their relative value to each other? Invariably, reasonable people will still disagree about which indicators should be used to measure comprehensive notions of development and wellbeing and whose values are reflected in a composite measure. In box 3, we outline an alternative wellbeing measure that attempts to tackle these challenges; this measure captures components similar to the HDI, without the HDI’s major limitations.

Box 3 **Developing an alternative wellbeing measure**

We sketch a proposal for constructing a wellbeing measure that is comparable to the HDI in what it captures without some of its shortcomings:

Alternative wellbeing measure=(GDP per capita)×(healthy life expectancy)×(median income)/(mean income)

This measure reflects expected lifetime income in good health. It captures the dimensions of income, health (including pollution, which reduces healthy life expectancy), and equality of opportunity (because in a more unequal society the difference between mean income and median income is higher, which weighs the measure down). This way of dealing with inequality of opportunity substitutes for the inclusion of schooling in the HDI.

Altogether the indicator would have the advantage of getting rid of the difficulty of aggregating fundamentally different things (income, life expectancy in years, and education). In addition, it has no upper limit by construction. Finally, and in contrast to HDI, this alternative measure has a straightforward interpretation.

## Beyond GDP: moving forward

We have summarised the arguments against using GDP, which gives a narrow picture of the value of better health and undervalues the true worth of health. We have also discussed a few alternatives, including the HDI and VLY, which can provide a fuller picture. No indicator is perfect. Despite its well known disadvantages, arguably few indicators have posed any considerable challenge to GDP’s hegemony. What hope is there for alternative metrics of development to gain mass popularity? We think it requires three things: repeated use of alternatives, policy leadership that values alternatives, and education supporting diffusion of the alternatives.

### Repeated use

Behavioural economics indicates that frequent reporting brings concepts to the “top of mind”—a psychological device based on repetition.^25^ The quarterly pronouncements of GDP by a national statistical bureau keep this metric in the forefront of people’s minds, so it needs a frequently reported challenger. One simple approach would be to announce other indicators of wellbeing alongside GDP every quarter. Perhaps a national authority could issue a quarterly report on life expectancy—one component of the HDI—which is currently reported only annually. Moreover, measuring life expectancy is arguably simpler and less data intensive than measuring GDP. Like GDP, life expectancy has a long time series.

### Policy leadership

**Fig 1:**
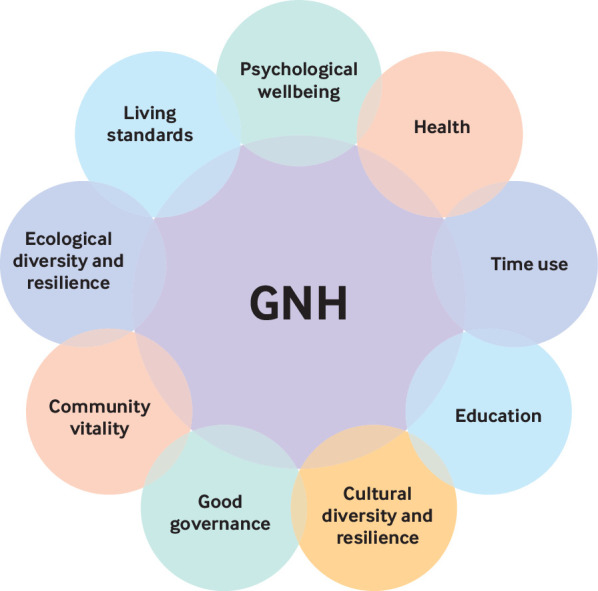
Bhutan’s Gross National Happiness (GNH) index

There are roles for national leadership, authority, and institutionalisation through changing laws, regulations, and rules. Some countries have attempted to counter dominance of GDP by developing their own measures. Bhutan, for example, developed Gross National Happiness (box 4, fig 1), spearheaded by its king.^26^

Box 4 **Bhutan’s happiness index**

Gross National Happiness (GNH) has nine domains (http://www.grossnationalhappiness.com/), each of which has 33 indicators that “are statistically reliable, are normatively important, and are easily understood by large audiences.”^27^ The indicators are weighted so that subjective, self reported indicators are assigned lower weights than objective, verifiable ones. The final index gives a score from 0 to 1 and is used to classify people into four happiness bands: deeply happy, extensively happy, narrowly happy, and unhappy. The most recent survey was conducted in 2015, surveying 8871 participants using a stratified four stage systematic random sampling design. Among the key findings, the survey found that urban populations tended to be happier than rural ones, men tended to be happier than women, and more educated people tended to be happier.^28^

### Education

An educated citizenry can help buffer media propagation of official GDP. We need to teach our children and the rest of society that accumulation of wealth and money is not everything. We need to show that a developed society in which citizens are educated with the freedoms and capabilities to pursue happiness, which are not necessarily at odds with national GDP growth but in support of it, is possible.

**Key messages**

Gross domestic product (GDP) per capita and its rate of growth are simple, important indicators of the current state of the economy and have been measured for a long time
But GDP per capita is a narrow, inadequate metric for capturing the true, full value of health investments
GDP should not be used as a standalone measure, but rather alongside complementary indicators of progress—such as “health income” or “value of life years,” which both capture the economic value of health improvements

## Notes

Contributors and sources: VYF is a health economist with a focus on health systems, financing, and incentives; DEB is an economist whose work focuses on health, demography, education, and labour; OO is a public health and health systems researcher with expertise in health economics and impact evaluation; KP’s research area is the analysis of the causes and consequences of long run economic growth; GY is a global health policy researcher who led the writing of the Commission on Investing in Health’s *Global Health 2035* report.. GY conceptualised the article. VYF wrote the first draft. VYF, OO, GY, DB, and KP revised the paper. GY is the guarantor.

This article is part of a series commissioned by *The BMJ*. Open access fees are paid for by the Bill and Melinda Gates Foundation, which had no involvement in the commissioning, peer review, or decision to publish.
